# Alterations in White Matter Network and Microstructural Integrity Differentiate Parkinson’s Disease Patients and Healthy Subjects

**DOI:** 10.3389/fnagi.2019.00191

**Published:** 2019-07-26

**Authors:** Nabin Koirala, Abdul Rauf Anwar, Dumitru Ciolac, Martin Glaser, Bogdan Pintea, Günther Deuschl, Muthuraman Muthuraman, Sergiu Groppa

**Affiliations:** ^1^Department of Neurology, University Medical Center of the Johannes Gutenberg University Mainz, Mainz, Germany; ^2^Centre for Biomedical Engineering, University of Engineering and Technology, Lahore, Pakistan; ^3^Department of Neurology, Institute of Emergency Medicine, Chisinau, Moldova; ^4^Laboratory of Neurobiology and Medical Genetics, State University of Medicine and Pharmacy “Nicolae Testemit̨anu”, Chisinau, Moldova; ^5^Department of Neurosurgery, University Medical Center of the Johannes Gutenberg University Mainz, Mainz, Germany; ^6^Department of Neurosurgery, Bergmannsheil Clinic, Ruhr University Bochum, Bochum, Germany; ^7^Department of Neurology, Christian-Albrechts-University, Kiel, Germany

**Keywords:** diffusion MRI, network connectivity analysis, Parkinson’s disease, aging, white matter

## Abstract

Parkinson’s disease (PD) is a neurodegenerative disease, neuropathologically characterized by progressive loss of neurons in distinct brain areas. We hypothesize that quantifiable network alterations are caused by neurodegeneration. The primary motivation of this study was to assess the specific network alterations in PD patients that are distinct but appear in conjunction with physiological aging. 178 subjects (130 females) stratified into PD patients, young, middle-aged and elderly healthy controls (age- and sex-matched with PD patients), were analyzed using 3D-T1 magnetization-prepared rapid gradient-echo (MPRAGE) and diffusion weighted images acquired in 3T MRI scanner. Diffusion modeling and probabilistic tractography analysis were applied for generating voxel-based connectivity index maps from each seed voxel. The obtained connectivity matrices were analyzed using graph theoretical tools for characterization of involved network. By network-based statistic (NBS) the interregional connectivity differences between the groups were assessed. Measures evaluating local diffusion properties for anisotropy and diffusivity were computed for characterization of white matter microstructural integrity. The graph theoretical analysis showed a significant decrease in distance measures – eccentricity and characteristic path length – in PD patients in comparison to healthy subjects. Both measures as well were lower in PD patients when compared to young and middle-aged healthy controls. NBS analysis demonstrated lowered structural connectivity in PD patients in comparison to young and middle-aged healthy subject groups, mainly in frontal, cingulate, olfactory, insula, thalamus, and parietal regions. These specific network differences were distinct for PD and were not observed between the healthy subject groups. Microstructural analysis revealed diffusivity alterations within the white matter tracts in PD patients, predominantly in the body, splenium and tapetum of corpus callosum, corticospinal tract, and corona radiata, which were absent in normal aging. The identified alterations of network connectivity presumably caused by neurodegeneration indicate the disruption in global network integration in PD patients. The microstructural changes identified within the white matter could endorse network reconfiguration. This study provides a clear distinction between the network changes occurring during aging and PD. This will facilitate a better understanding of PD pathophysiology and the direct link between white matter changes and their role in the restructured network topology.

## Introduction

Parkinson’s disease (PD) is a neurodegenerative disorder characterized by motor dysfunctions including, among others, tremor, difficulty in initiating and executing voluntary movements (akinesia/freezing/bradykinesia) and muscular rigidity (Caligiore et al., 2016). These deficits are believed to arise from the dysfunction within the dopaminergic system and alterations in the integrity of distributed brain neural networks (Brooks and Pavese, 2011; Koirala et al., 2016). A positive modulation of this network might be critical for therapeutic efficacy. Hence, studying the network properties devised by these anatomical connections between the brain regions offers a valuable framework for exploration of a multi-systemic disease like PD and identification of relevant pathological phenotypes.

Diffusion tensor imaging (DTI) provides information regarding anatomical connections in the brain by measuring the diffusivity of water molecules in brain tissues (Basser et al., 1994). The variability in water diffusion in different directions is most commonly quantified by measures such as fractional anisotropy (FA), mean diffusivity (MD), axial diffusivity (AD) and radial diffusivity (RD) (Bodini and Ciccarelli, 2009; Scholz et al., 2009). As an effective and non-invasive tool to investigate microstructural alterations in white matter, DTI’s use in research of neurodegenerative diseases has escalated in recent years (Acosta-Cabronero et al., 2010; Douaud et al., 2011). Previous DTI studies have shown that the diffusivity measures could be reliably used in PD and related disorders to explore the microstructure either at the level of whole brain (Hattori et al., 2012) or in regions of interest (ROI) such as the substantia nigra (Chan et al., 2007; Peran et al., 2010). Usually the derived connectivity model using DTI contains a large number of anatomical connection patterns. The precise description of these anatomical connectivity patterns could be simplified by characterizing it within the network analysis framework. A graph theoretical approach provides a powerful tool to analyse complex networks within this framework by representing the regions of the brain in terms of nodes and anatomical connections in terms of edges (Bullmore and Sporns, 2009), ultimately quantifying the network properties using various parameters (Fleischer et al., 2017; Koirala et al., 2017).

Recent studies on brain network topology using diffusion magnetic resonance imaging (dMRI) have shown increased network connectivity in prodromal PD and decreased local connectivity in de novo PD, disclosing regional reorganization of nodes in PD patients compared to healthy subjects (Wen et al., 2017). Similarly, in another study the PD patients not yet exposed to dopaminergic therapy showed disrupted structural connectivity within several motor and non-motor regions, supporting the presence of disconnectivity in those networks (Nigro et al., 2016). A widespread structural network disruption involving basal ganglia and the frontotemporoparietal region was observed in PD patients with mild cognitive impairment (MCI), however, no significant differences were found between healthy controls and PD patients without MCI (Galantucci et al., 2017). Furthermore, network reconstruction from diffusion data of PD patients has demonstrated a widespread structural disconnectivity, which could be potentially linked to motor and non-motor symptoms of PD patients (Shah et al., 2017).

When analyzed using graph theoretical measures, the modular organization of structural networks was found to be different between healthy adults and elderly population (Wu et al., 2012). Older subjects exhibited lower global efficiency and higher local clustering in comparison to younger subjects (Zhu et al., 2012). Moreover, a longitudinal network analysis revealed that the structural brain networks obtained from T1-anatomical images develop a localized organization pattern with substantial alterations in older ages (Wu et al., 2013). Concomitant studies in healthy subjects have reported microstructural white matter changes with normal aging. White matter tract analysis using dMRI has shown significant changes in FA and MD between adults and elderly within all fascicles (Yeatman et al., 2014). Furthermore, FA values were found to be increased during adulthood reaching a peak at 20–42 years of age and then decreasing, while MD followed the inverse trend – first decreasing, reaching the minimum at 18–41 years of age and then increasing later in life (Lebel et al., 2012). Therefore, in this study, we evaluated the alterations of structural network connectivity in PD patients and healthy subjects of different age in order to reveal the differences in microstructural integrity and network architecture between the diseased state and normal aging. The observed brain network alterations in various regions along with the microstructural brain changes might be a valuable tool for the differentiation of PD patients from healthy subjects in the future and moreover provide an unprecedented opportunity to observe the brain network rearrangement during PD progression.

## Materials and Methods

### Data Acquisition

In total, 178 subjects were included in this study. The data for 38 subjects were acquired at the Kiel University, Kiel (Germany). These subjects were divided into three groups: young healthy controls (HC_Y_) (*n* = 13; mean age ± SD 30.63 ± 4.34 years), middle-aged healthy controls (HC_M_) (*n* = 13; 53.15 ± 6.98 years) and 12 idiopathic PD patients (PD) [66.75 ± 7.18 years, mean Unified Parkinson’s Disease Rating Scale (UPDRSIII) score 34.5 ± 8.4_[Med_ _OFF]_ and 14.30 ± 14.46_[med_ _ON]_, mean L-dopa equivalent dose 621.69 ± 394.85 mg, mean disease duration 13.6 ± 6.5, H and Y 3.8 ± 0.8_[Med_ _OFF]_ and 2.7 ± 0.5_[Med_ _ON]_]. Demographic and group distribution details are shown in Table 1. For every participant, MRI was acquired using a 3 Tesla MRI scanner (Philips Achieva) with an 8 channel head coil. The whole-brain DTI scans were obtained with a voxel resolution of 2 × 2 × 2 mm^3^ covering a field of view of 224 × 224 × 120 mm. These diffusion-weighted images were acquired for 32 directions with a *b* value of 1000 s/mm^3^, echo time (TE) of 59 ms and repetition time (TR) of 11855 ms. Also, 60 contiguous slices with fat saturation “on” and 5 b_0_ images were obtained for each acquisition. Additionally, high resolution T1 images were obtained using standard MPRAGE (magnetization-prepared 180 degrees radio-frequency pulses and rapid gradient-echo) sequence with TR = 7.7 ms, TE = 3.6 ms and flip angle = 8°. These scans consisted of 160 contiguous sagittal slices with a voxel resolution of 1 × 1 × 1 mm^3^ and a field of view of 224 × 224 × 170 mm. The study protocol used was approved by the local ethics committee in Kiel and all patients signed a written informed consent regarding the procedure.

**TABLE 1:** Demographics and group distribution.

**Dataset**	**Group**	**N (Male/female)**	**Mean age (years)**
Kiel	Young healthy controls (HC_Y_)	13 (4/9)	30.63 ± 4.34
	Middle-aged healthy controls (HC_M_)	13 (8/5)	53.15 ± 6.98
	Parkinson’s disease patients (PD)	12 (4/8)	66.75 ± 7.18
NKI	Middle-aged healthy controls – NKI (HC_M–NKI_)	70 (14/56)	53.71 ± 3.11
	Elderly healthy controls – NKI (HC_E–NKI_)	70 (18/52)	66.20 ± 2.53

*The table shows the demographical and group characteristics of the subjects included in the study. NKI, Nathan Kline Institute.*

As a control experiment, an additional 140 age- and sex-matched (with middle-aged healthy and PD group from Kiel dataset) healthy subjects were obtained from a publicly available dataset from the Nathan Kline Institute (NKI)^1^ and fed into the same analytical framework. These healthy subjects were classified into two subgroups according to age as middle-aged healthy controls – NKI (HC_M–NKI_) (*n* = 70; 53.71 ± 3.11 years) and elderly healthy controls – NKI (HC_E–NKI_) (*n* = 70; 66.20 ± 2.53 years). Demographics are detailed in Table 1. In this cohort, whole-brain DTI scans were obtained using a 3T MRI scanner (Siemens Magnetom Trio Tim) with 32 channel head coil and 2 × 2 × 2 mm^3^ voxel resolution. For each subject, 64 contiguous diffusion weighted slices were obtained for 137 directions with *b* value of 1500 s/mm^3^ and TE and TR of 85 ms and 2400 ms, respectively. High resolution T1 images (176 slices) using standard MPRAGE sequence were also obtained for each subject with TE = 2.52 ms, TR = 1900 ms and voxel resolution of 1 × 1 × 1 mm^3^.

### Data Analysis

**FIGURE 1:**
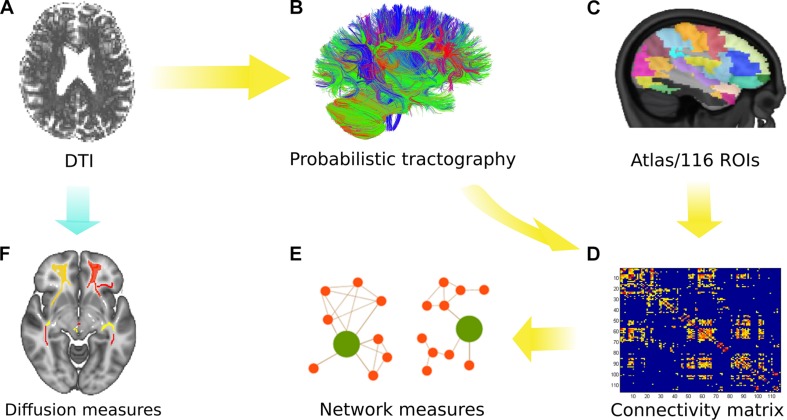
Overview of the analytical approach. **(A)** Diffusion tensor imaging (DTI) was performed with all subjects. **(B)** Probabilistic tractography was used to obtain the connectivity matrix using the AAL atlas (116 ROIs) as depicted in **(C)**. **(D)** The connectivity matrix was generated depicting the probability from a seed to a target region (for 116 regions). **(E)** Network measures were obtained from the group comparison. **(F)** Microstructural analysis was performed using various diffusion measures identified after tract-based spatial statistics (TBSS) and permutation inference.

The MRI scans primarily obtained in DICOM format were converted into NIfTI format using the built-in MRIcron toolbox (Rorden, 2012). Then the obtained NIfTI images were pre-processed using the FDT tool in FSL (Behrens et al., 2003a,b, 2007). The complete processing protocol is explained in detail elsewhere (Groppa et al., 2014) and is depicted in Figure 1. In brief, by using FSL^2^, microstructural analysis was performed in order to obtain FA, MD, AD, and RD. During pre-processing, images were first corrected for motion artifacts and eddy currents by applying an affine transformation, and then the brain extraction tool was used to remove the non-brain tissue (Smith, 2002).

For network analysis, the connectivity matrices were obtained for each subject using the seed masks for 116 ROIs defined by the Automated Anatomical Labeling atlas (Tzourio-Mazoyer et al., 2002). The links or the entries in the connectivity matrix represent the ratio between the number of samples (or streamlines) that pass through the ROI (j) and all generated streamlines from the ROI (i). This weighted connectivity index between the ROIs in the matrix was then analyzed using global and local network parameters obtained via the Brain Connectivity Toolbox (Rubinov and Sporns, 2010)^3^. The acquired undirected and weighted measures were used to compute the network parameters of distance, clustering and centrality to observe the influence over global information transfer, principle hubs, and reorganization for segregation or integration. The detailed formulation and definitions for the network parameters are explained elsewhere (Rubinov and Sporns, 2010). Finally, a two-sample Kolmogorov–Smirnov (KS) test followed by the Bonferroni–Holm (B–H) method for multiple comparison correction was performed to reveal significant (*p* < 0.05) differences in network measures between the groups.

Network-based statistic (NBS) analysis^4^ was used to assess differences in the interregional connectivity matrices between the groups. NBS analysis deals with the multiple comparisons problem posed by connectomic data by evaluating the null hypothesis at the level of interconnected subnetworks rather than individual connections (Zalesky et al., 2010). Here, the weighted undirected connectivity matrices obtained from probabilistic tractography were subjected to NBS analysis to identify the difference between the groups. A threshold of the *t*-statistic greater than 3 was determined using the effect size (Cohen’s d) and was used for individual connections and 5000 permutations were generated to build up the null distribution. Finally, the networks showing impaired connectivity between regions when compared to other groups obtained with significance of *p* < 0.05 were reported. Further details regarding the procedure are mentioned elsewhere (Korgaonkar et al., 2014).

In order to study the microstructural white matter changes, tract-based spatial statistics (TBSS) method was applied (Smith et al., 2006). The FMRIB58_FA_1 mm standard space image provided by FSL was used as the target for the nonlinear registration and the skeleton threshold was set to 0.2 (Woolrich et al., 2009; Jenkinson et al., 2012). For statistical analyses of the diffusion measures (FA, MD, RD, and AD), permutation test (using the randomize tool) (Winkler et al., 2014) was applied between the three subject groups to test six different contrasts (HC_Y_ >/< PD, HC_M_ >/< PD, HC_Y_ >/< HC_M_). For the contrast HC_y_-PD and HC_M_-PD, age and sex were used as the nuisance variable and only sex for the contrast HC_y_–HC_M_. For each contrast, 5000 permutations were carried out with different thresholding options [voxel-based, cluster-based and threshold-free cluster enhancement (TFCE)]. Family-wise error (FWE, *p* < 0.05) correction was applied to account for multiple comparisons. In this study, TFCE was preferred over voxel-based thresholding and cluster-based thresholding due to its significant benefits of overcoming the problem of defining the cluster-forming threshold while keeping intact the sensitivity benefits (Smith and Nichols, 2009).

## Results

### Network Analysis

The compared groups did not differ significantly (*p* > 0.05) in terms of sex but differed significantly (*p* < 0.001) in terms of age.

### Network Topology in Healthy Subjects

**FIGURE 2:**
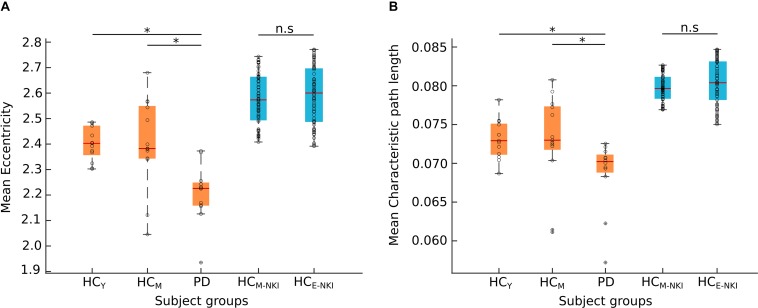
Network measures. The boxplots shows the mean eccentricity **(A)** and mean characteristic path length **(B)** obtained from both cohorts. The Nathan Kline Institute (NKI) dataset of healthy control groups (shown here in blue) showed no significant difference (n.s) between middle-aged healthy controls (HC_M–NKI_) and elderly healthy controls (HC_E–NKI_). The young (HC_Y_) or middle-aged (HC_M_) healthy controls and Parkinson’s disease (PD) patients from Kiel cohort (shown here in orange) showed significant difference in both network measures. Significance was tested using a two-sample Kolmogorov–Smirnov test with the Bonferroni-Holm method for multiple comparisons correction. ^*^Characteristic path length: HC_Y_-PD (*p* = 0.0013), HC_M_-PD (*p* = 0.0264); eccentricity: HC_Y_-PD (*p* = 0.0242), HC_M_-PD (*p* = 0.0275). The black circles in the plot represent the data points.

No significant differences (*p* > 0.05) in network parameters were found between the two healthy control groups from the main cohort (HC_Y_ and HC_M_). In the control analysis performed using the NKI dataset, we as well observed no significant (*p* > 0.05) differences between the two healthy control groups – HC_M–NKI_ (λ_mean_ = 0.0798 ± 0.0017, *E*_mean_ = 2.5771 ± 0.1023) and HC_E–NKI_ (λ_mean_ = 0.0805 ± 0.0029, *E*_mean_ = 2.5897 ± 0.1138) as shown in Figure 2.

From NBS analysis no significant differences were obtained from the comparison between the healthy control groups from either dataset used in the study.

### Network Topology in PD Patients

Both characteristic path length and eccentricity were found to be significantly decreased (*p* = 0.0013 and *p* = 0.0242, respectively, B–H corrected) in PD patients (λ_mean_ = 0.068 ± 0.004, *E*_mean_ = 2.205 ± 0.114) when compared to HC_Y_ (λ_mean_ = 0.073 ± 0.002, *E*_mean_ = 2.401 ± 0.065) and HC_M_ (λ_mean_ = 0.072 ± 0.005, *E*_mean_ = 2.403 ± 0.16) as shown in Figure 2. Hence, confirming that the obtained network changes are specific to PD pathology and are independent of age.

**FIGURE 3:**
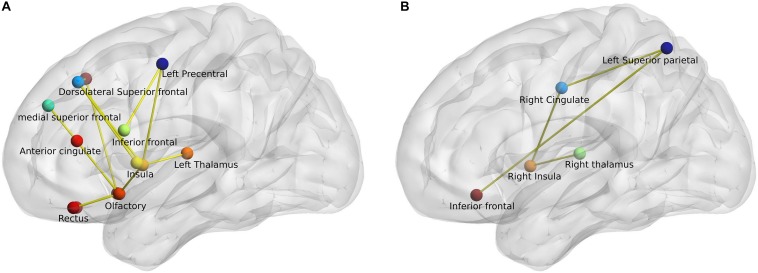
Network-based statistic-derived subnetworks. **(A)** Left sagittal view shows the significant subnetwork obtained from the comparison between young healthy controls (HC_Y_) and PD patients. **(B)** Left sagittal view shows the significant subnetwork obtained from the comparison between middle-aged healthy controls (HC_M_) and PD patients. The circles represent the nodes and lines represent the edges or connections with a *t*-stat > 3 and *p* < 0.05 (corrected).

Network-based statistic analysis (*t*-stat > 3, *p* < 0.05, corrected for multiple comparisons) revealed a network of anatomical regions with a significantly decreased connectivity profile in PD patients when compared to both healthy control groups. The analysis revealed 13 regions with significantly diminished connectivity strength in PD patients when compared with young healthy controls including bilateral superior and inferior frontal, anterior cingulate, olfactory cortex, gyrus rectus, insula, and left precentral and thalamus. Similarly, the comparison between middle-aged healthy controls (HC_M_) and PD patients (HC_M_ > PD) generated 5 regions – bilateral inferior frontal, right cingulate gyrus, thalamus, insula, and left superior parietal region, as depicted in Figure 3.

## Microstructural Analysis

### Microstructural Integrity in Healthy Subjects

**FIGURE 4:**
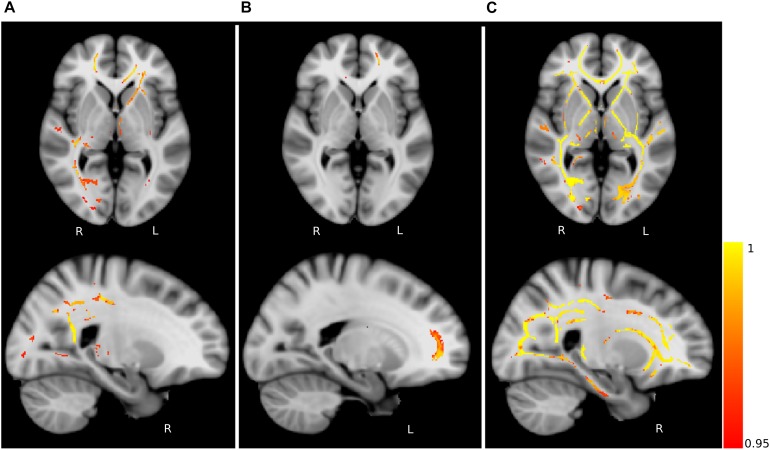
Fractional anisotropy (FA) differences. Axial and sagittal slices showing significant differences between **(A)** young and middle-aged healthy controls (HC_Y_ > HC_M_), **(B)** middle-aged healthy controls and PD patients (HC_M_ > PD) and **(C)** young healthy controls and PD patients (HC_Y_ > PD). The slices show the threshold-free cluster enhancement (TFCE) thresholded results (*p* < 0.05, FWE corrected) superimposed on the FSL’s 1 mm MNI152 standard brain. The scale [1 – *p*-value] represents the significance of the clusters with yellow indicating higher and red indicating lower statistical significance. L and R denote the left and right hemisphere of the brain.

The compared healthy control groups did not differ significantly (*p* > 0.05) in terms of sex. The comparison between the healthy subjects (HC_Y_ > HC_M_) revealed only one significant cluster of FA difference in the right anterior corona radiata (Figure 4 and Table 2). No differences in AD, MD, or RD between the healthy control groups were identified. These findings highlight the differences in microstructural integrity occurring in healthy controls (as a consequence of physiological aging processes) in comparison to PD patients (as a consequence of the disease).

**TABLE 2:** Details of significant clusters.

**Contrast**	**FA_max_**	**Voxels**	**COG X _(mm)_**	**COG Y _(mm)_**	**COG Z _(mm)_**	**Anatomical region**
HC_Y_ > HC_M_	0.991	403	108	173	74.7	Forceps minor
HC_M_ > PD	1	22702	87.7	110	92.7	Fornix
HC_Y_ > PD	1	64242	87.8	115	88.4	Fornix

*The table contains the characteristics of the dominant clusters shown in Figure 4. The first row shows the characteristics of the cluster obtained from the contrast between young and middle-aged healthy controls (HC_*Y*_ > HC_*M*_), second and third rows show the characteristics from the contrast between middle-aged healthy controls and PD patients (HC_*M*_ > PD) and between young healthy controls and PD patients (HC_*Y*_ > PD), respectively. The FA_*max*_ indicates the maximum fractional anisotropy (FA) value in the cluster, the column “Voxels” contains the number of voxels in the cluster and “COG” shows the coordinates of the center of gravity in the cluster. The anatomical region indicates the region with coordinates of COG according to JHU (Johns Hopkins University) white matter atlas in FSL.*

### Microstructural Alterations in PD Patients

Group-wise FA comparison of PD patients and middle-aged healthy controls (PD < HC_M_) showed significant (*p* < 0.05, FWE corrected, controlled for sex and age) differences in splenium, body and tapetum of corpus callosum, and fornix. For the comparison of PD and young healthy subjects (PD < HC_Y_), the clusters were identified in splenium of corpus callosum, left corticospinal tract and corona radiata. All significant clusters are shown in Figure 4 and the detailed characteristics of the clusters are outlined in Table 2.

**FIGURE 5:**
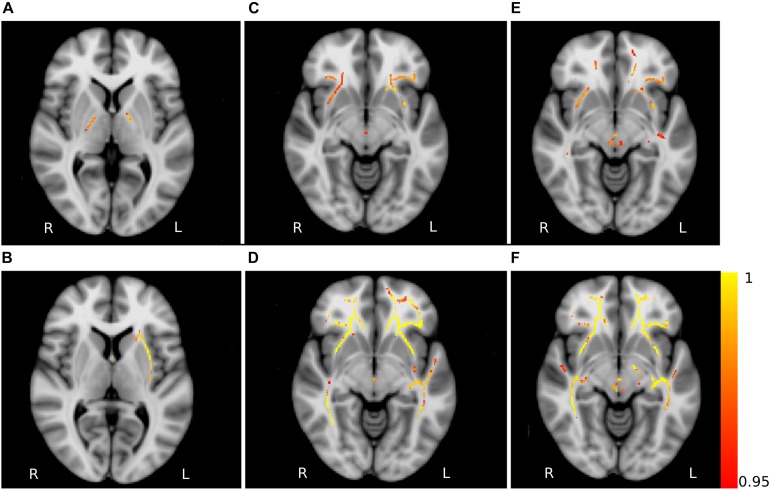
Non-FA diffusivity measures differences. Axial slices of non-FA measures: axial diffusivity obtained for the contrasts HC_Y_ > PD **(A)** and HC_Y_ < PD **(B)**, mean diffusivity for the contrasts HC_M_ < PD **(C)** and HC_Y_ < PD **(D)**, radial diffusivity for the contrasts HC_M_ < PD **(E)** and HC_Y_ < PD **(F)**. The slices show the results after the threshold free cluster enhancement (TFCE) thresholding (*p* < 0.05, FWE corrected), superimposed on the FSL’s 1 mm MNI152 standard brain. The scale [1 – *p*-value] represents the significance of the clusters with yellow indicating higher and red indicating lower statistical significance. L and R denote the left and right hemisphere of the brain.

The analysis of AD showed significant clusters between PD patients and young healthy controls in the posterior limb of internal capsula (PD < HC_Y_) and right external capsula (PD > HC_Y_). Similarly, a significant group difference in MD was detected between PD patients and young healthy subjects (PD > HC_Y_) in splenium and body of corpus callosum, external capsula, anterior, and superior corona radiata and fornix. Differences in MD values between the PD patients and middle-aged healthy controls (PD > HC_M_) were identified in external capsula, anterior and superior corona radiata and splenium of corpus callosum. Furthermore, the group-wise RD analysis between PD and middle-aged healthy controls (PD > HC_M_) identified significant differences predominantly in the body of corpus callosum and fornix and between PD patients and young healthy controls (PD > HC_Y_) in external capsula, the body of corpus callosum, fornix and the posterior thalamic radiation. The clusters obtained in AD, MD and RD analysis are depicted in Figure 5.

## Discussion

**FIGURE 6:**
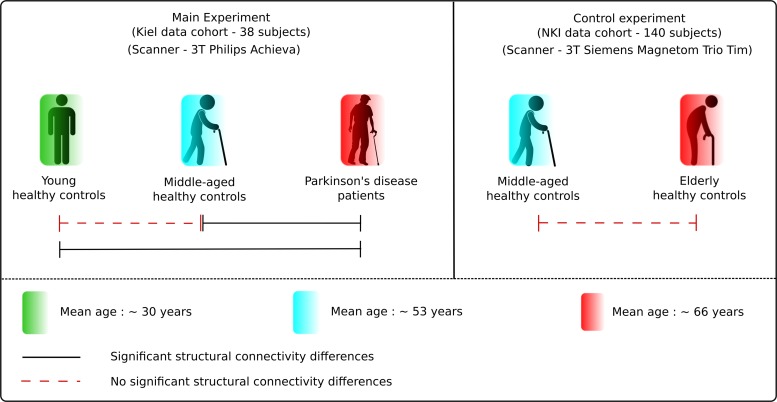
Results summary. The figure illustrates the main results obtained in the study. There was no significant difference between the healthy subject groups but significant differences between the healthy and PD patients group, presenting the clear delineation in aging and neurodegeneration.

The results obtained demonstrate specific network alterations in patients with PD that are beyond physiological aging. The network connectivity analysis showed a significant difference in eccentricity and characteristic path length between PD patients and age- and sex-matched healthy controls. Both network measures were significantly reduced in PD patients in comparison to young and middle-aged healthy controls. Crucially, these network patterns did not differ between young, middle-aged and elderly healthy subjects (Figure 6). Hence, these findings clearly delineate the divergence in microstructural and network topological changes occurring during PD-dependent neurodegeneration.

Characteristic path length is the average shortest distance between the regions in the network and is a measure of global integration. Eccentricity is defined as the maximal shortest path length between any two regions in the network and is a measure of centrality, which denotes the importance of a region in the network (Olde Dubbelink et al., 2014). Structural connections are fundamental to allow the neural elements to coordinate their activity into coherent dynamic states. To achieve such coherent dynamics, structural networks shape the information flow between local brain regions to accomplish segregation by forming local network communities or integration by enabling global communication between communities (Sporns, 2013).

### Network Alterations in PD

The distance parameters evaluated here, namely characteristic path length and eccentricity, show the alterations in the structural network, which in turn provides valuable details about the information flows and network integration. In the case of characteristic path length, a measure of global integration, the shorter the path length the “more effective” the information flow (Sporns, 2011). Hence, the observed overall decrease in the presence of shorter paths within the networks in PD patients in comparison to healthy control groups clearly highlights the disruption of information flow within the networks. Moreover, this disruption was further supported by an overall decrease in eccentricity, an indicator of the importance of a region in the network, in the PD networks, reflecting an impediment to efficient network integration. We previously showed that the eccentricity in PD patients correlated inversely with the applied voltage during deep brain stimulation for optimal clinical response (Koirala et al., 2017). Hence, the eccentricity does not only differentiate the diseased group from healthy controls but also reflects the amount of network abnormality, accentuating its impact on network efficiency and stressing the overall importance of distance measures. Furthermore, in agreement with previous studies using functional MRI that show a decrease in global efficiency in PD patients in comparison to healthy controls (Skidmore et al., 2011) and also a notable loss of long-range connections in PD patients with mild cognitive impairment (Baggio et al., 2014), the observed decrease in the eccentricity and characteristic path length in PD patients in our study clearly indicates a disruption of global network integration and information transfer.

Network-based statistic analysis demonstrated specific networks of reduced connectivity mirroring the white matter structural abnormalities seen in PD patients when compared to healthy controls. In particular, the identified networks included the regions from motor and sensorimotor, basal ganglia and limbic systems that are primary involved in PD. As expected, the number of significantly different regions in the network comparison between young healthy subjects and PD patients was higher than for middle-aged healthy subjects and PD patients, corresponding to the microstructural alterations obtained from the same contrasts. Previous studies using NBS for PD patients showed significant differences in the connections primarily involved in key components of the limbic system, basal ganglia and sensorimotor areas (Nigro et al., 2016; Li et al., 2017). Here in this study, we further complement those findings by showing specific basal ganglia – frontal networks when compared to healthy controls.

### Network Alterations in Aging

Previous functional MRI studies on the reorganization of brain networks in aging have shown that the default mode network is the most compromised one during the process of aging (Jie et al., 2014; Sala-Llonch et al., 2015). Hence, the anatomical regions found in the network when comparing young healthy subjects and PD patients shows some overlapping regions for aging, which vanish when compared with the middle-aged healthy controls. In addition, the overlap of the brain networks during development and disorders has been previously discussed (Douaud et al., 2014), which further highlights the importance of network metrics for the differentiation between aging- or PD-specific processes. The structural abnormalities occurring in PD patients in frontal, parietal and cingulate cortex have been shown using both white matter (Vercruysse et al., 2015) and gray matter analysis (Beyer et al., 2007; Rektorova et al., 2014; Mak et al., 2015). Hence, the differences in connectivity properties obtained from these regions using NBS analysis indicate that the white matter networks play an important pathophysiological role in PD. Moreover, no differences were identified in the comparison between the healthy subject groups in either cohort, which strongly demonstrates that the detected network changes are not the result of aging but rather derive from PD pathology.

### Microstructural Alterations in PD

Several previous studies reported that the reduced FA in olfactory cortex and gyrus rectus explains the dysfunction within the olfactory system and white matter tracts connecting it to other brain regions, causing olfactory impairment that is present even in the premotor phase of the disease (Ibarretxe-Bilbao et al., 2010; Doty, 2012). The insula is known to be an integrating node for multiple brain networks and its involvement in PD, particularly in relation to the non-motor symptoms, was repeatedly stipulated (Christopher et al., 2014; Criaud et al., 2016). Although diffusivity properties obtained from DTI are indirect measures of white matter integrity, it could be speculated that the PD-related neurodegenerative mechanisms (e.g., alpha-synuclein-driven damage to the presynaptic terminals) may impair axonal transport and consequently produce axonal degeneration (Nigro et al., 2016). The microstructural analysis performed in our study revealed white matter alterations linked to the disrupted network topology in PD patients. Lower FA values were identified in PD patients in the body, splenium and tapetum of the corpus callosum, fornix, and corona radiata – the fiber tracts known from previous studies to be affected in PD and considered primary substrates of cognitive impairment (Matsui et al., 2006; Zheng et al., 2014). These fiber tracts were also detected in the networks obtained from the NBS analysis, further indicating that the observed network alterations potentially cause the disconnection phenomena within the white matter.

Moreover, the significant alterations in MD observed between PD patients and healthy controls are in line with previous studies addressing the diffusion alterations associated with PD (Hall et al., 2016). An increase in RD is an indicator of increased demyelination and axonal degeneration (Alexander et al., 2011), and it has been shown that in PD the unmyelinated axons are more vulnerable to degeneration than myelinated axons (Orimo et al., 2011). Hence, the clusters of higher RD values in PD patients could bring axonal degeneration in PD patients to the foreground.

### Microstructural Integrity in Aging

There were only minimal white matter changes between the young and middle-aged healthy control groups, which could be the result of insignificant variations in the white matter volume occurring between the ages of 30–55 (Sowell et al., 2003), the age interval of the healthy controls from our study. More importantly, not all of the regions of FA differences, detected from the comparison between PD patients and healthy controls were identified between healthy subject groups, which further reinforces that microstructural patterns of aging and neurodegeneration are distinct. It is known that AD alterations relate to the variation in axonal integrity within different brain regions during the life span (Kumar et al., 2013). Here, the differences in AD in PD patients in comparison to young healthy subjects were detected in more regions than shown in Kumar et al. (2013) for physiological aging. Thus, we further speculate that the decrease in AD might indicate neurodegeneration while an increase reflects compensation.

Overall, we found that network distance parameters serve as a novel approach for a non-invasive investigation of the emerging PD pathology. The performed analyses are substantiated by regional microstructural alterations showing an involvement at the anatomical level with predilection sites for the impact of PD pathology. The differential network reorganization pattern in PD compared to physiological aging facilitates the understanding of network modifications in PD pathophysiology. The primary motivation of this study was to provide the distinction between the network changes during aging and due to PD in order to demonstrate specific neurodegenerative network and microstructural alterations that occur in PD, which are beyond physiological aging. Hence, the use of different age groups in the study strengthens this objective. In one limitation of our study, we used the aged matched healthy controls dataset obtained from an open source, to control for the network changes due to physiological aging. As a result, the mean network measures obtained for two cohorts differ marginally but consistently (see Figure 2). As shown in the previous studies the connectivity values based on probabilistic tractography vary upon DTI directions (Vaessen et al., 2010), different scanner (Owen et al., 2013), applied density threshold (Andreotti et al., 2014) or different approach of obtaining the measures (Bonilha et al., 2015). We assume that the difference in our study is also due to the difference in DWI sequence and the scanning site. Furthermore, it would be very interesting to see the relationship with these network outcomes and structural changes with the neuropsychological data to associate these network topological disruptions with clinical outcomes. However, for the clinical implementation of the studied methodology, future studies with higher sample size, homogeneous data acquisition and correlation to the clinical parameters should be investigated.

## Conclusion

The network connectivity analysis revealed significant differences in distance measures, indicating the disruption of network integration in PD patients that can underlie the network topological differences observed during the disease condition and normal aging. The NBS, demonstrating differences mainly in frontal and cingulate regions between the PD and healthy controls, further emphasizes the observed network alterations. Diffusion alterations within the white matter tracts evidenced by microstructural analysis highlight the structural underpinnings of the disrupted network topology. Our results contribute to the understanding of the PD pathophysiology regarding the white matter integrity and its role in the network maintenance, and can be used in the current field of biomarker research for a better differentiation of PD patients from healthy elderly and middle-aged subjects.

## Ethics Statement

This study was carried out in accordance with the recommendations of “Ethik-Kommission der Medizinischen Fakultät der Christian-Albrechts-Universität zu Kiel” with written informed consent from all subjects. All subjects gave written informed consent in accordance with the Declaration of Helsinki. The protocol was approved by the “Ethik-Kommission der Medizinischen Fakultät der Christian-Albrechts-Universität zu Kiel.”

## Author Contributions

NK performed the measurement, carried out the analysis, and wrote the first draft of the manuscript. AA and DC contributed to the measurement and its organization, and conceptual direction for the analysis. MG, BP, and GD reviewed the draft and experimental procedure for critique comments and suggestions. MM and SG contributed to the conception and execution of the study and the critical review of the manuscript. All authors discussed the manuscript and agreed to the final version.

## Conflict of Interest Statement

The authors declare that the research was conducted in the absence of any commercial or financial relationships that could be construed as a potential conflict of interest.

## Footnotes

1 http://fcon_1000.projects.nitrc.org/indi/enhanced/

2 http://www.fmrib.ox.ac.uk/fsl/

3 https://sites.google.com/site/bctnet/

4 https://sites.google.com/site/bctnet/comparison/nbs
